# Iron-deficiency anemia in relation to body mass index among Iraqi primigravida women

**DOI:** 10.25122/jml-2023-0066

**Published:** 2023-06

**Authors:** Ekhlas Jabbar Kadhim

**Affiliations:** 1 Department of Obstetrics and Gynecology, College of Medicine, University of Baghdad, Baghdad, Iraq

**Keywords:** body mass index, breastfeeding, iron-deficiency anemia, iron status, primigravida women

## Abstract

Anemia affects approximately half a billion women of reproductive age worldwide, with 31% of pregnant women in Iraq aged 15–49 years experiencing anemia. This condition is associated with increased risks of maternal and fetal morbidity and mortality, including stillbirths, miscarriages, prematurity, and low birth weight. This study investigated the correlation between iron-deficiency anemia (IDA) and body mass index (BMI) among primigravidae in Iraq. One hundred primiparous women in their third trimester were recruited from Baghdad Medical City Teaching Hospital and Teaching Hospital of Obstetrics and Gynecology in Karbala. Participants were categorized into four groups based on BMI: underweight (BMI < 18.5 kg/m^2), normal weight (BMI 18.5–24.9 kg/m^2), overweight (BMI 25–29.9 kg/m^2), and obese (BMI ≥ 30 kg/m^2). Demographic and medical history data were collected from the participants, and hematological investigations were conducted to measure hemoglobin (Hb), packed cell volume (PCV), mean corpuscular volume (MCV), and serum ferritin levels. Statistical significance was determined at p<0.05. The study enrolled 100 primiparous women, including 10 underweight, 24 normal weight, 28 overweight, and 38 obese participants. Analysis revealed a significant decrease in Hb levels among obese individuals compared to the normal weight group. Moreover, a significant difference in serum ferritin levels was observed between the obese and the other three groups (underweight, normal weight, and overweight). The findings indicated an inverse correlation between high BMI and serum ferritin and Hb levels. The study concluded that anemia is more common in obese pregnant women compared to normal-weight women. Furthermore, it demonstrates varying trends of iron-deficiency anemia (IDA) in relation to the body mass index (BMI) of pregnant women.

## INTRODUCTION

Anemia is one of the most common medical disorders encountered during pregnancy worldwide, affecting both developing and developed countries. It is characterized by a decrease in the number and/or size of erythrocytes or a reduction in hemoglobin levels, impairing the transportation of oxygen through the blood vessels. Anemia is considered an indicator of malnutrition and inadequate health and is a common health problem that adversely affects woman and their offspring [1]. Approximately half a billion women of reproductive age are affected by anemia, with 29% of non-pregnant and 38% of pregnant women worldwide aged 15-49 years experiencing anemia. The etiology of anemia varies and include factors such as malnutrition (e.g., folate, riboflavin, vitamins A and B12 deficiencies) and infectious diseases like malaria, tuberculosis, and HIV [2].

According to World Health Organization (WHO), the prevalence of iron-deficiency anemia (IDA) in the Eastern Mediterranean Region among pregnant women aged 15–49 years is 38.9% (3.9 million), with 0.1% (1.1 million) experiencing severe anemia. In Iraq, the prevalence of anemia among pregnant women aged 15-49 years is 31%, with 0.4% having severe anemia (blood hemoglobin concentration below 70 g/L) [3]. Anemia in pregnant women is associated with increased risks of maternal and fetal mortality and complications such as stillbirths, miscarriages, prematurity, and low birth weight. Women with IDA often experience symptoms such as difficulty breathing and sleeping, fatigue, cardiac arrhythmia, and fainting, and are more susceptible to infectious diseases, bleeding, and pre-eclampsia. They also report post-partum cognitive disorder and post-partum depression [4]. Infants born to mothers with IDA are at risk of low birth weight, intrauterine growth retardation, prematurity, and increased mortality rates. IDA also has a profound impact on fetal growth during the first trimester, with an increased risk of premature labor [5]. In the third trimester, infants may experience mental developmental abnormalities, cognitive and emotional delays, as well as delays in language and motor development. Breastfeeding may also be affected as iron levels in breast milk decline over time during lactation [6].

Iron deficiency anemia rates have been found to increase more than fivefold from the beginning of pregnancy to delivery. Research studies have consistently shown a significant decrease in hemoglobin levels as pregnancy progresses, resulting in a rise in the prevalence of anemia. Specifically, the rates of anemia at different stages of pregnancy have been reported as follows: 1.9% during weeks 0-13, 13.4% during weeks 14-25, 17.7% during weeks 26-36, and 21.9% during weeks 37-42 [7]. Recent studies showed that the prevalence of obesity among pregnant is a significant risk factor for the mother and the fetus [8]. Obese women are at a higher risk of experiencing complications such as macrosomia, stillbirth, neural tube defects, preeclampsia, gestational diabetes mellitus, premature labor, and caesarian delivery than women with lower body mass index (BMI) [9]. Furthermore, research has indicated a significant relationship between high BMI and iron deficiency, with several studies demonstrating the impact of BMI on iron homeostasis [10-13].

The demand for iron supplementation increases in the first trimester for individuals with a high BMI due to a negative correlation with iron status. In the third trimester, the average daily need for iron supplementation averages close to 1g per day, 300 mg of which is delivered to the fetus and the placenta (this amount is necessary and crosses the placenta to the fetus regardless of the iron status of the mother). Around one-fifth of the amount of iron is eliminated through the gastrointestinal tract, kidney, and elementary system, while the remainder is utilized to synthesize maternal hemoglobin. This increase in demand exceeds the iron reserves of most women, meaning that pregnant women should deplete 20 to 48 mg of iron supplements daily. Vegetarian diets, which provide only around 13mg of iron daily, may not meet the increased iron needs during pregnancy, potentially leading to anemia and iron deficiency if adequate supplementation is not taken [14]. According to WHO recommendations, daily iron supplementation in pregnant individuals is advised, with a dosage ranging from 30 to 60mg of elemental iron. This corresponds to 60mg of elemental iron in the form of 300mg ferrous sulfate, 180mg ferrous fumarate, or 500mg ferrous gluconate [15]. This study aimed to investigate the distribution of iron-deficiency anemia in relation to body mass index among Iraqi primiparous pregnant women and assess the correlation between maternal BMI and iron status.

## MATERIAL AND METHODS

The present study employed an analytic cross-sectional design to collect data using a convenient sampling method. Verbal consent was obtained from 100 women who met specific criteria, including being anemic, primiparous, in the third trimester (28th to 36th weeks), and having a singleton pregnancy. Participants were recruited from the antenatal care units of two hospitals, Baghdad Medical City Teaching Hospital and Teaching Hospital of Obstetrics and Gynecology in Karbala. Women with other conditions, such as hypertension or diabetes, were excluded from the study.

Hematological investigations were performed to assess various parameters, including mean corpuscular volume (MCV), hemoglobin (Hb), packed cell volume (PCV), and serum ferritin (SF) levels. Anemia was defined as a hemoglobin level below 11g/dL, MCV less than 80 mg/dL (indicating microcytic and hypochromic red blood cells), and serum ferritin levels below 30 µg/L [16, 17]. Height and weight measurements were recorded, and the body mass index (BMI) was calculated using the mathematical formula: weight in kilograms divided by the square of height in meters. The participants were then classified into four BMI categories according to the Institute of Medicine (IOM) recommendations: underweight (<18.5), normal weight (18.5–24.9), overweight (25–29.9), and obese (including all classes) (≥30).

## STATISTICAL ANALYSIS

Statistical analysis was conducted using SPSS software, version 22.0. The data were expressed as means (M) and standard deviations (SD). One-way analysis of variance (ANOVA) was employed to compare the BMI groups, and Pearson's correlation coefficient was calculated to evaluate the correlations between variables. A significance level of p<0.01 was considered statistically significant.

## RESULTS

A total of 100 primigravidae women were enrolled in this study. The demographic characteristics of the study participants are summarized in Table 1.

**Table 1:** General characteristics of the 100 enrolled women

Variable	Underweight	Normal weight	Overweight	Obese	Total	p value
Mean (SD)						
Age	23.2 (2.5)	21.4 (2.9)	21.1 (3.1)	21.1(3.1)	21.4 (3.05)	0.271
Gestational Age	33.2 (3.8)	32.8 (2.8)	32.3 (3.04)	32.7(3.01)	32.7 (3.03)	0.86
HB	8.8 (0.6)	8.7 (0.8)	8.09 (0.9)	8.02 (0.8)	8.3 (0.9)	0.001
MCV	73.7 (2.21)	74.8 (2.21)	72.3 (9.5)	74.2 (5.3)	73.7 (5.3)	0.361
PCV	23.8 (2.8)	26.1 (2.2)	24.3 (2.5)	25.02(2.07)	24.9 (2.4)	0.18
Serum ferritin	28.2 (0.7)	25.2 (4.5)	25.2 (2.4)	22.5 (1.3)	24.4 (3.2)	0.000
Number (%)	10 (10%)	24 (24%)	28 (28%)	38 (38%)	100	

The mean age was 21.4 (SD=3.05) years, and the mean gestational age was 32.7 (SD=3.03) weeks. Among the participants, 10% were underweight, 24% were of normal weight, 28% were overweight, and 38% were obese. Table 1 also presents the distribution of anemia among the different BMI categories. The results revealed no significant difference in hematocrit (PCV) and mean corpuscular volume (MCV) among the different BMI groups. However, a significant decrease in hemoglobin (HB) concentration and serum ferritin (SF) levels was observed in obese women compared to those of normal weight, as shown in Table 1.

Table 2 shows the multiple comparisons between different BMI groups. The analysis revealed that the difference in HB levels was significant only between the normal-weight and obese groups (p=0.001). On the other hand, a significant difference in SF levels was observed between all BMI groups, indicating that obese women were more likely to have iron deficiency compared to underweight, normal-weight, and overweight women. Furthermore, the results of the study indicated a negative association between BMI and Hb (-0.318) and serum ferritin (SF) (-0.485) levels, suggesting lower iron status with increasing BMI (Table 3).

**Table 2:** Multiple comparisons between different groups

Dependent Variable	(I) BMI. Real	(J) BMI. Real	Mean Difference (I-J)	Std. Error	Sig.
HB g/100ml	underweight	normal weight	.04417	.32888	.893
		overweight	.75071	.32189	.022
		obese	.81368	.31055	.010
	normal weight	underweight	-.04417	.32888	.893
		overweight	.70655*	.24306	.005
		obese	.76952*	.22782	.001
	overweight	underweight	-.75071	.32189	.022
		normal weight	-.70655*	.24306	.005
		obese	.06297	.21762	.773
	obese	underweight	-.81368	.31055	.010
		normal weight	-.76952*	.22782	.001
		overweight	-.06297	.21762	.773
SF gm/100ml	underweight	normal weight	3.20000*	1.02149	.002
		overweight	2.93571*	.99980	.004
		obese	5.67105*	.96456	.000
	normal weight	underweight	-3.20000*	1.02149	.002
		overweight	-.26429	.75495	.727
		obese	2.47105*	.70762	.001
	overweight	underweight	-2.93571*	.99980	.004
		normal weight	.26429	.75495	.727
		obese	2.73534*	.67593	.000
	obese	underweight	-5.67105*	.96456	.000
		normal weight	-2.47105*	.70762	.001
		overweight	-2.73534*	.67593	.000

* . Correlation is significant at the 0.05 level (2-tailed).

**Table 3:** Pearson’s correlation coefficient

		BMI	SF	HB
	Pearson Correlation	1	-.485**	-.318**
BMI	Sig. (2-tailed)		.000	.001
	N	100	100	100
	Pearson Correlation	-.485**	1	.203*
SF g/100ml	Sig. (2-tailed)	.000		.042
	N	100	100	100
	Pearson Correlation	-.318**	.203*	1
HB g/100ml	Sig. (2-tailed)	.001	.042	
	N	100	100	100

** . Correlation is significant at the 0.01 level (2-tailed).

Pearson’s Correlation coefficient exhibited a significant negative correlation between Hb, BMI, and serum ferritin (-0.48) and (-0.31), respectively, as shown in Table 3.

## DISCUSSION

The current study revealed a significant association between body mass index (BMI) and iron deficiency anemia in pregnant women. The findings demonstrated that overweight and obese pregnant women had a higher prevalence of iron deficiency anemia compared to those with normal weight (Table 1), and this may be explained by the low grade of inflammation that often present in obese patients that can impact iron uptake and metabolism through elevation of hepcidin levels. The study also showed a negative correlation between BMI and serum ferritin and Hb. The correlation between high BMI and iron deficiency anemia was demonstrated in various populations, including children, adolescents, and pregnant women [18-23].

Recent studies that focused on iron homeostasis in obese women revealed disturbances in iron metabolism, including absorption and distribution, ultimately leading to iron deficiency. These disturbances have been attributed to the overexpression of hepcidin, an iron regulatory protein synthesized in the liver [24,25]. Elevated hepcidin levels associated with obesity can lead to inflammation and negatively impact iron uptake and metabolism [26]. Furthermore, hepcidin levels tend to increase from the first trimester to the third trimester of pregnancy, which may contribute to the observed association between BMI and anemia [27]. However, this remains only a hypothesis and needs further studies to confirm it. Other studies have reported lower rates of anemia in overweight and obese women from Sudanese populations compared to other racial groups [22-23].

This study has limitations, including its small sample size and potential biases associated with the sampling method and estimation of hepcidin levels. Future research with larger sample sizes and more comprehensive assessments is needed to better understand iron deficiency anemia in our specific population.

## CONCLUSION

The study emphasizes the importance of providing pregnant women with comprehensive nutritional education regarding the significance of iron-rich food sources and the impact of dietary choices on iron absorption. Addressing misconceptions, such as the belief that a high-carbohydrate diet alone is sufficient during pregnancy, is essential. Gynecologists and healthcare providers should actively monitor hemoglobin levels in pregnant women, considering their BMI, to detect and manage iron deficiency anemia effectively.

## References

[1] Department of Nutrition for Health and Development, World Health Organization Anemia Policy Brief.

[2] Stevens G, Finucane M, De-Regil L, Paciorek C (2013). Global, regional, and national trends in hemoglobin concentration and prevalence of total and severe anemia in children and pregnant and non-pregnant women for 1995–2011: a systematic analysis of population-representative data. LancetGlob Health.

[3] WHO (2015). The global prevalence of anaemia in 2011.

[4] Allen LH (2000). Anemia and iron deficiency: effects on pregnancy outcome. Am J Clin Nutr.

[5] Abu-Ouf NM, Jan MM (2015). The impact of maternal iron deficiency and iron deficiency anemia on child’s health. Saudi Med J.

[6] Chang S, Zeng L, Brouwer ID, Kok FJ, Yan H (2013). Effect of Iron Deficiency Anemia in Pregnancy on Child Mental Development in Rural China. Pediatrics.

[7] Messenger H, Lim B (2016). The Prevalence of Anemia In Pregnancy In A Developed Country, How Well Understood Is It?. J-Preg Child Health.

[8] Heslehurst N, Ells LJ, Simpson H, Batterham A (2007). Trends in maternal obesity incidence rates, demographic predictors, and health inequalities in 36,821 women over a 15-year period. BJOG.

[9] Leddy MA, Power ML, Schulkin J (2008). The Impact of Maternal Obesity on Maternal and Fetal Health. Rev Obstet Gynecol.

[10] Pinhas-Hamiel O, Newfield RS, Koren I, Agmon A (2003). Greater prevalence of iron deficiency in overweight and obese children and adolescents. Int J Obes Relat Metab Disord.

[11] Sánchez A, Rojas P, Basfi-Fer K, Carrasco F (2016). Micronutrient Deficiencies in Morbidly Obese Women Prior to Bariatric Surgery. Obes Surg.

[12] Zhao L, Zhang X, Shen Y, Fang X (2015). Obesity and iron deficiency: a quantitative meta-analysis. Obes Rev.

[13] Garcia-Valdes L, Campoy C, Hayes H, Florido J (2015). The impact of maternal obesity on iron status, placental transferrin receptor expression and hepcidin expression in human pregnancy. Int J Obes (Lond).

[14] Gautam CS, Saha L, Sekhri K, Saha PK (2008). Iron Deficiency in Pregnancy and the Rationality of Iron Supplements Prescribed During Pregnancy. Medscape J Med.

[15] World Health Organization (2012). Guideline: Daily iron and folic acid supplementation in pregnant women.

[16] Api O, Breyman C, Çetiner M, Demir C, Ecder T (2015). Diagnosis and treatment of iron deficiency anemia during pregnancy and the postpartum period: Iron deficiency anemia working group consensus report. Turk J Obstet Gynecol.

[17] Leveno KJ (2013). Williams Manual of Pregnancy Complications.

[18] Moayeri H, Bidad K, Zadhoush S, Gholami N, Anari S (2006). Increasing prevalence of iron deficiency in overweight and obese children and adolescents (Tehran Adolescent Obesity Study). Eur J Pediatr.

[19] Nead KG, Halterman JS, Kaczorowski JM, Auinger P, Weitzman M (2004). Overweight children and adolescents: a risk group for iron deficiency. Pediatrics.

[20] Shi Z, Lien N, Kumar BN, Dalen I, Holmboe-Ottesen G (2005). The sociodemographic correlates of nutritional status of school adolescents in Jiangsu Province, China. J Adolesc Health.

[21] Eftekhari MH, Mozaffari-Khosravi H, Shidfar F (2009). The relationship between BMI and iron status in iron-deficient adolescent Iranian girls. Public Health Nutr.

[22] Abbas W, Adam I, Rayis DA, Hassan NG, Lutfi MF (2017). A Higher Rate of Iron Deficiency in Obese Pregnant Sudanese Women. Open Access Maced J Med Sci.

[23] Hemamalini J (2013). Anemia in Relation to Body Mass Index and Waist Circumference among Andhra Pradesh Women. J Obes Weight Loss Ther.

[24] Mujica-Coopman MF, Brito A, López de Romaña D, Pizarro F, Olivares M (2015). Body mass index, iron absorption and iron status in childbearing age women. J Trace Elem Med Biol.

[25] del Giudice EM, Santoro N, Amato A, Brienza C (2009). Hepcidin in obese children as a potential mediator of the association between obesity and iron deficiency. J Clin Endocrinol Metab.

[26] Andrews M, Soto N, Arredondo-Olguín M (2015). Association between ferritin and hepcidin levels and inflammatory status in patients with type 2 diabetes mellitus and obesity. Nutrition.

